# Inequalities in the prevalence of diabetes mellitus and its risk factors in Sri Lanka: a lower middle income country

**DOI:** 10.1186/s12939-018-0759-3

**Published:** 2018-04-17

**Authors:** Ambepitiyawaduge Pubudu De Silva, Sudirikku Hennadige Padmal De Silva, Rashan Haniffa, Isurujith Kongala Liyanage, Saroj Jayasinghe, Prasad Katulanda, Chandrika Neelakanthi Wijeratne, Sumedha Wijeratne, Lalini Chandika Rajapaksa

**Affiliations:** 1 Department of Community Medicine, Faculty of Medicine, University of Colombo, Colombo, Sri Lanka; 2 Centre for Tropical Medicine, University of Oxford, Oxford, UK; 3 Department of Para Clinical Sciences, Faculty of Medicine, General Sir John Kotelawala University, Colombo, Sri Lanka; 4 Department of Clinical Medicine, Faculty of Medicine, University of Colombo, Colombo, Sri Lanka; 5 Department of Obstetrics & Gynaecology, Faculty of Medicine, University of Colombo, Colombo, Sri Lanka

**Keywords:** Inequalities, Diabetes mellitus, Risk factors of diabetes mellitus, Lower middle income country

## Abstract

**Background:**

Explorations into quantifying the inequalities for diabetes mellitus (DM) and its risk factors are scarce in low and lower middle income countries (LICs/LMICs). The aims of this study were to assess the inequalities of DM and its risk factors in a suburban district of Sri Lanka.

**Methods:**

A sample of 1300 participants, (aged 35–64 years) randomly selected using a stratified multi-stage cluster sampling method, were studied employing a cross sectional descriptive design. The socioeconomic indicators (SEIs) of the individual were education level and occupational category, and at the household level, the household income, social status level and area deprivation level. DM was diagnosed if the fasting plasma glucose was ≥126 and a body mass index (BMI) of > 27.5 kg/m^2^ was considered high. Asian cut-off values were used for high waist circumference (WC). Validated tools were used to assess the diet and level of physical activity. The slope index of inequality (SII), relative index of inequality (RII) and concentration index (CI) were used to assess inequalities.

**Results:**

The prevalence of DM and its risk factors (at individual or household level) showed no consistent relationship with the three measures of inequality (SII, RII and CI) of the different indices of socio economic status (education, occupation, household income, social status index or area unsatisfactory basic needs index).

The prevalence of diabetes showed a more consistent pro-rich distribution in females compared to males. Of the risk factors in males and females, the most consistent and significant pro-rich relationship was for high BMI and WC. In males, the significant positive relationship with high BMI for SII ranged from 0.18 to 0.35, and RII from 1.56 to 2.25. For high WC, the values were: SII from 0.13 to 0.27 and RII from 1.9 to 3.97. In females the significant positive relationship with high BMI in SII ranged from 0.13 to 0.29, and RII from 2.3 to 4.98. For high WC the values were: SII from 028 to 0.4 and RII 1.99 to 2.39.

Of the other risk factors, inadequate fruit intake showed a consistent significant pro-poor distribution only in males using SII (− 0.25 to − 0.36) and in both sexes using CI. Smoking also showed a pro-poor distribution in males especially using individual measures of socio-economic status (i.e. education and occupation).

**Conclusions:**

The results show a variable relationship between socioeconomic status and prevalence of diabetes and its risk factors. The inequalities in the prevalence of diabetes and risk factors vary depending on gender and the measures used. The study suggests that measures to prevent diabetes should focus on targeting specific factors based on sex and socioeconomic status. The priority target areas for interventions should include prevention of obesity (BMI and central obesity) specifically in more affluent females. Males who have a low level of education and in non-skilled occupations should be especially targeted to reduce smoking and increase fruit intake.

## Background

Diabetes mellitus (DM) is a major non-communicable disease contributing to mortality, morbidity and other non-communicable diseases. There were approximately 171 million adults with diabetes mellitus worldwide in the year 2000 and this is projected to rise to 366 million by 2030 [1]. The highest percentage of increase (> 100%) of diabetes mellitus prevalence is observed in low income countries/lower middle income countries while high income countries experience an increase of 54% [1]. The diabetes burden in low income countries/lower middle income countries is double that of high income countries for all age groups [1]. Among the South Asian region India, Pakistan and Bangladesh are within the top ten countries projected to have the highest numbers of adults with diabetes mellitus for 2000 and 2030 [1].

Although the excess global mortality of diabetes mellitus is reported to be 5.2% of all deaths it causes significant debilitation with its complications and associations with other non-communicable diseases [2, 3]. Unlike other non-communicable diseases that cause acute mortality, diabetes mellitus may persist for a lengthy period with the complications causing a heavy economic burden to the state [4]. The life expectancy loss due to diabetes mellitus has been estimated to be less than 7.5 years among those with diabetes mellitus compared to their non-diabetes equivalents [5, 6]. A person with diabetes mellitus lives with the disease, requiring treatment for its control and complications, thereby incurring heavy costs for nations [7]. A person with diabetes mellitus has 2.3 times the medical expenditure of a person without the disease and account for more than 1 in 5 health care dollars in the USA [8]. The economic loss in the African region ranges from $2144.3 to $11,431.6 per diabetes case per year [9].

Due to its high burden and economic cost it is imperative that diabetes mellitus is prevented. Towards this end community screening for the presence of disease, its complications and risk factors, and life style modification programs in the primary health care setting have been introduced. However the alarming increase in the prevalence of this disease and its complications in low income countries/lower middle income countries demonstrates the failure of primary and secondary prevention measures in these settings [10, 11]. Although technical and medical solutions such as disease control and medical care within the health sector are important they alone are not sufficient. Improvements in living and working conditions and access to known medical solutions, would lead to dramatic reductions in the inequalities of the disease. Addressing the social determinants of health can yield greater and sustainable returns. Action on social determinants of health empowers people, communities and countries and empowerment is a potent method to change both social structure and conditions. Also action on the social determinants of health will not only improve individual health but also will indicate that society has moved towards meeting human needs and ensuring rights. The advantages of a social determinant of the health approach are: it bridges the artificial distinction between technical and social interventions; it seeks to redress the imbalance between curative and preventive action, individualized and population-based interventions and act on structural conditions in society. Thus a social determinants approach offers a better hope for sustainable and equitable outcomes [12].

The lack of a social determinants approach in the preventive strategies employed may have contributed to the failure [12] of current approaches to tackle DM. In order to develop a social determinants approach it is necessary to assess the inequalities in the distribution of diabetes mellitus and its risk factors.

Most studies on social determinants of health are from high-income countries and they demonstrate health inequalities in the distribution of the disease and risk factors with higher prevalence rates of the disease observed in the poor and marginalized. Studies from the European continent have demonstrated inequalities in the prevalence of DM and its complications due to socioeconomic position with lower socio-economic communities being more affected compared to higher socio-economic groups [13–17]. Many of these studies use the Slope Index of Inequality (SII) and the Relative Index of Inequality (RII). Evidence from lower middle income settings suggests that poverty is associated with higher diabetes incidence and inequality of diabetes care [18, 19]. Although surveys in South Asia describe higher prevalence of DM associated with higher economic status, these studies do not quantify inequality [20–25].

In Sri Lanka the incidence of DM and its risk factors show a steady rise and a positive association with economic status [26–31]. Interestingly all surveys on DM and its risk factors in Sri Lanka do not describe the distribution in the plantation sector. The plantation sector comprises of all plantations which are 20 acres or more in extent and ten or more resident laborers and came into being during the colonial period. It comprises the lowest socioeconomic category and comparatively poor infrastructure. The population distribution in urban, rural and plantation sectors in Sri Lanka are approximately 18.3%, 77.3% and 4.4% respectively [32]. The depth and severity of poverty are also the highest among estate sector in Sri Lanka as shown by a head count index 32%, poverty gap index 6.2% and poor households 25.8%. Individuals living in estate communities experience a variety of economic and social constraints, including short falls in access to productive assets like land and water, gap in physical infrastructures like power, transport and communications, imperfectly functioning product and input markets, inadequate technology and weak institutional arrangements. The health inequalities are more marked in the plantation sector with basic health indictors such as maternal mortality rate and infant mortality rates being higher compared to others [32]. We have studied a representative sample from all three sectors and reported the social gradients observed in the prevalence of diabetes mellitus in Sri Lanka [33]. This report examined social gradient of the prevalence of diabetes and its risk factors across different socio-economic strata using chi square test for trend [33]. This method is best suited in situations where there is a linear ascending or descending trend or gradient in a health outcome, across socio-economic status. Therefore, in the present analysis we explore inequalities in diabetes prevalence and its risk factors using other indices the SII, the RII and the concentration index. We compare these across individual, household and area level socioeconomic indices and its variability for diabetes mellitus and its selected risk factors.

## Methods

A detailed description of the study methods has been published in 2012 [33, 34]. A cross sectional design with stratified multistage cluster sampling was used to randomly select 1300 adults between the ages of 35 to 64 years from the Kalutara district, which comprises of urban, rural and plantation sectors. A sample size of 1300 was reached calculated based on 16% prevalence of DM, with a margin of error at 3%, α error at 5%, dropout rate of 10% and cluster effect of 2. In order to produce a wider scatter of the sample the cluster size was limited to 20 as people of similar socioeconomic status tend to cluster together. The level of stratifications was at the urban, rural and plantation sectors with the Grama Niladari Division (GND) (GND the lowest village level administrative division in Sri Lanka) being the primary sampling unit. GNDs were randomly selected, probability proportionate to the size of its population of 35 to 64 years age group. Within each GND 20 households were randomly selected using the electoral registry and a single eligible individual was randomly selected from each selected household. Information was gathered using validated questionnaires administered by trained data collectors. Anthropometric measurements and laboratory investigations for fasting plasma glucose were also conducted.

We used the following definitions for the categorization of study participants; participants were categorized as suffering from DM if they were on insulin or hypoglycaemics within the past four weeks or if they had a fasting plasma glucose of ≥126 mg/dl [35]; they were categorized as having an impaired glucose tolerance if they had fasting plasma glucose of ≥100 mg/dl and < 126 mg/dl [35]; a body mass index of > 27.5 kg/m^2^ was considered high while waist circumferences of ≥90 cm and ≥ 80 cm were regarded as high for men and women respectively [36–38]. A presence of a family history of DM was deemed when parents or siblings were known to have diabetes mellitus. A culturally adapted version of the International Physical Activity Questionnaire validated for Sri Lanka [39] was used to assess the level of physical activity. It assesses vigorous, moderate and mild physical activities carried out during the past week and classifies the subjects into insufficiently active, sufficiently active and highly active categories. The quality of diet was assessed with a tool developed and validated for Sri Lanka [39]. The tool grouped fruit and sugar intake in the diet, based on a scoring system described by Arembepola [39]. Those who consumed at least one glass of alcohol during the past fortnight were classified as alcohol users while those who smoked at least once during the past fortnight were classified as smokers.

The highest level of education attained was recorded. Occupation was classified as mentioned by the Registrar General in Britain and adapted to the local setting [40]. Monthly household income was obtained in local currency (Sri Lankan Rupees). Social status index (SSI) was assessed as described by De Silva [40] and the Unsatisfactory Basic Needs Index (UBNI) as described by Satharasinghe [41].

Analysis was conducted with STATA 13. All results presented were weighted and standardized for age and sex of the Sri Lankan population.

The SII, RII and concentration index were employed to measure health inequality [42–44]. SII and RII are regression–based indexes used to illustrate the magnitude of socioeconomic position as a source of inequalities in health. The approach involves calculating the mean health status of each socioeconomic group and then ranking classes by their socioeconomic status (not by their health). SII and RII reflect the socioeconomic dimension to inequalities in health. SII measures the absolute effect while RII measures relative inequality. We calculated SII and RII as described by Schneider et al. [44].

SII (rate difference) is slope of the regression line estimated by the weighted least square method and represents the change in measured outcome event when the position of the socioeconomic status changes by one unit.

The approach for SII is creating a weighted sample of the whole population which is ranked from the most disadvantaged subgroup (at rank 0) to the most advantaged (at rank 1) according to, selected socioeconomic indicator (eg. level of education or income). The population of each socioeconomic category is considered in terms of its range in the cumulative population distribution, and the midpoint of this range. The health indicator of interest is regressed against this midpoint value for selected socioeconomic indicator wealth or education subgroups using an appropriate model. The predicted values of the health indicator are calculated for the two extremes (rank 1 and rank 0). The difference between the predicted values at rank 1 and rank 0 (covering the entire distribution) generates the slope index of inequality value. The slope index of inequality (SII) is then defined as the slope of the regression line showing the relationship between a class’s health status and its relative rank (R,) in the socioeconomic distribution. It can be interpreted as the absolute effect on health of moving from the lowest socioeconomic group through to the highest.

RII (rate ratios) can be estimated in two ways: one way is to divide the SII by the mean level of population health or by the frequency of the health problem in the population, the other way is to divide the predicted value of the regression at the highest point (range = 1) by the predicted value of the regression at the lowest point (range = 0), The second method for the RII is calculated by log-linear or logistic regression after the logarithmic r logit transformation of the dependent variable.

Because SII is an absolute measure, it is sensitive to changes in the mean level of population health or changes in the frequency of the health problem being studied. If the mean level of health increases in the same proportion in all the socioeconomic categories, the SII will increase, whereas the relative differences remain constant.

The concentration index is a bivariate measure, which uses the distribution of a health variable and a variable describing the socioeconomic standards against which the distribution is to be assessed [45]. This accounts for both the strength of the association and the magnitude of differences between health variable and relative rank in the socioeconomic distribution. This index is based on the ”concentration curve” which plots the cumulative percentage of the health variable (y axis) against the cumulative percentage of the population, ranked by socioeconomic level, beginning with the poorest, and ending with the richest (x-axis). The concentration index is defined as twice the area between the concentration curve and the line of equality (the 45-degree line). The index lies between − 1 and 1. When there is no socioeconomic-related inequality, the concentration index is zero. The concentration index can be computed for good health as well as ill health. The index has a negative value when the curve lies above the line of equality, indicating disproportionate concentration of the health variable among the poor, and a positive value when it lies below the line of equality.

Ethics approval was received from the Ethics Review Committee of Faculty of Medicine, University of Colombo (Reference Number: EC/08/119). Written informed consent was obtained from all study participants.

## Results

The age and sex adjusted diabetes mellitus prevalence was 14.7% from a representative sample in Sri Lanka and the distribution of the study sample and its representativeness has been described previously [33]. The socio-demography of the study population by fasting glucose levels is described in Table 1 [33]. Using the same database we calculated the SII, RII and concentration index for diabetes and its risk factors on individual education, individual occupation, household level income, household level SSI, and UBNI, which is an area level deprivation index.

**Table 1:** Socioeconomic determinants of fasting glucose levels among adults in Kalutara, Sri Lanka

Characteristic	Glucose tolerance (*n* = 1234)						*p*
	Normal (*n* = 832)		IFG (*n* = 200)		DM (*n* = 202)		
	Number	Percent	Number	Percent	Number	Percent	
Sex							
Male	437	75.7%	93	10.2%	98	14.1%	*0.236*
Female	395	66.6%	107	18.2%	104	15.2%	
Age category							*< 0.001* ^a^
35 to 39 Years	152	84.6%	23	10.6%	11	4.8%	
40 to 44 Years	164	74.7%	28	13.0%	29	12.3%	
45 to 49 Years	142	71.4%	36	12.6%	39	16.0%	
50 to 54 Years	124	62.9%	38	18.6%	40	18.6%	
55 to 59 Years	136	58.0%	47	18.5%	43	23.4%	
60 to 64 Years	114	66.9%	28	14.4%	40	18.7%	
Ethnicity							
Sinhalese	554	71.2%	130	14.4%	156	14.4%	
Tamil	208	60.6%	50	10.4%	23	29.0%	
Muslim	69	68.5%	19	11.6%	22	20.0%	*0.003*
Other	01	91.5%	01	3.3%	01	05.2%	
Sector							
Urban	224	58.6%	68	17.8%	90	23.6%	
Rural	397	69.9%	83	14.6%	88	15.5%	*0.001* ^a^
Plantation	211	74.3%	49	17.3%	24	08.5%	
Education category							
No schooling	35	73.8%	07	9.8%	06	16.4%	
Grade 5 or below	199	76.5%	53	10.9%	31	12.6%	
Grade 6 to 10	274	70.5%	56	13.4%	78	16.1%	*0.019* ^a^
O/L to Grade 12	190	72.9%	49	15.3%	45	11.8%	
AL and above	91	66.9%	25	15.8%	31	17.3%	
Occupation category							
Professional	07	97.5%	02	1.0%	03	1.4%	
Technical & clerical	38	72.3%	11	15.3%	12	12.4%	
Vendors & sellers	82	60.0%	23	14.3%	31	25.6%	*0.175*
Skilled manual workers	160	84.8%	24	7.9%	21	7.2%	
Unskilled manual workers	181	74.8%	36	14.3%	23	10.8%	
Retired	35	60.2%	15	19.7%	14	20.1%	
Unemployed	40	70.6%	8	6.5%	11	22.9%	
Housewife	257	65.5%	76	18.1%	82	16.4%	
Income Category (per month)							
< 10,000	293	73.2%	66	12.4%	53	14.3%	
10,000 to 30,000	440	70.3%	107	14.5%	113	15.1%	*0.005* ^a^
> 30,000	74	64.7%	23	19.2%	35	16.1%	
Social status index							
1st quintile	158	66.3%	38	16.3%	47	17.4%	
2nd quintile	153	71.1%	39	14.5%	51	14.5%	
3rd quintile	158	73.2%	39	12.0%	45	14.7%	*0.006* ^a^
4th quintile	163	71.7%	38	14.7%	42	13.6%	
5th quintile	182	76.1%	43	16.9%	17	7.0%	
UBNI							
1	80	91.0%	07	4.7%	08	4.3%	
2	119	93.6%	34	1.4%	13	5.0%	
3	176	70.8%	44	16.9%	28	12.3%	*< 0.001* ^a^
4	214	71.6%	46	13.6%	64	14.7%	
5	243	63.3%	69	16.0%	89	20.7%	

^a^chi square test for trend was used. *IFG*: Impaired Glucose Tolerance, *DM*: Diabetes Mellitus, *IFG*: Impaired Fasting Glucose; *UBNI*: Unsatisfactory Basic Needs Index

Inequalities in the prevalence of diabetes varied across gender and depending on the measures used. Using SII (Table 2) showed a significant and consistent positive relationship between prevalence of diabetes in females and different measures of inequalities at individual, household and area-wise deprivation (i.e. increases in prevalence with higher income or higher social status). At the individual level the SII of occupations were significant, though in opposite directions both sexes (i.e. higher prevalence seen with ‘lower’ strata of occupations). In males individual level of education and household income showed a pro-rich distribution for prevalence of diabetes.

**Table 2:** Comparison of the slope index of inequality for diabetes mellitus and its risk factors among males and females by different measures of socio economic statuses

Morbidity / risk factor	Individual		Household		Area
	Education (95%CI)	Occupation (95%CI)	Income (95%CI)	Social Status Index (95%CI)	Unsatisfactory Basic Needs Index (95%CI)
Male					
Prevalence of diabetes mellitus	0.12 (0.01–0.22)*	− 0.15 (− 0.2- -0.01)*	0.16 (0.05–0.27)*	0.02 (− 0.08–0.11)	0.08 (− 0.01–0.17)
Prevalence of impaired glucose tolerance	0.1 (− 0.01–0.21)	− 0.2 (− 0.38- − 0.02)*	0.05 (− 0.08–0.17)	-0.02 (− 0.13–0.09)	0.07 (− 0.03–0.18)
High body mass index	0.35 (0.21–0.49)*	− 0.31 (− 0.5- -0.13)*	0.25 (0.11–0.4)*	0.18 (0.05–0.31)*	0.27 (0.14–0.4)*
High waist circumference	0.27 (0.15–0.38)*	− 0.34 (− 0.5- -0.18)*	0.28 (0.16–0.39)*	0.13 (0.01–0.25)*	0.23 (0.12–0.34)*
Family history of diabetes mellitus	0.17 (0.04–0.31)*	0.06 (− 0.15–0.27)	0.08 (− 0.06–0.21)	0.13 (0.02–0.25)*	0.27 (0.15–0.39)*
Insufficient physical activity	0.02 (− 0.11–0.16)	−0.20 (− 0.4–0.001)	0.09 (− 0.05–0.23)	0.001 (− 0.12–0.12)	0.36 (−0.08–0.16)
Inadequate fruit intake	−0.36 (− 0.49- -0.23)*	0.4 (0.18–0.61)*	− 0.33 (− 0.47- -0.18)*	− 0.25 (− 0.37- -0.13)*	− 0.32 (− 0.44- -0.21)*
Inappropriate sugar intake	0.01 (− 0.12–0.14)	− 0.1 (− 0.31–0.1)	0.08 (− 0.07–0.022)	0.02 (− 0.11–0.14)	0.03 (−0.1–0.15)
Smoking	−0.18 (− 0.32- -0.03)*	0.34 (0.11–0.56)*	− 0.17 (− 0.33- − 0.12)*	-0.12 (− 0.25–0.01)	0.01 (− 0.12–0.15)
Alcohol consumption	0.01 (− 0.13–0.16)	0.08 (− 0.14–0.29)	0.05 (− 0.1–0.2)	−0.11 (− 0.23–0.1)	−0.22 (− 0.34- -0.1)*
Female					
Prevalence of diabetes mellitus	0.14 (0.2–0.25)*	− 0.34 (− 0.61- -0.07)*	0.13 (0.02–0.24)*	0.21 (0.11–0.31)*	0.29 (0.2–0.38)*
Prevalence of impaired glucose tolerance	0.01 (−0.12–0.15)	0.15 (− 0.13–0.43)	0.07 (− 0.07–0.21)	0.04 (− 0.07–0.15)	0.05 (− 0.06–0.16)
High body mass index	0.37 (0.23–0.52)*	− 0.54 (− 0.8–0.28)	0.23 (0.08–0.38)*	0.41 (0.29–0.52)*	0.5 (0.38–0.62)*
High waist circumference	0.37 (0.22–0.51)*	− 0.56 (− 0.81- -0.31)*	0.28 (0.13–0.43)*	0.35 (0.23–0.47)*	0.4 (0.28–0.53)*
Family history of diabetes mellitus	0.15 (0.02–0.28)*	− 0.43 (− 0.73- -0.12)*	0.09 (− 0.04–0.22)	0.17 (0.07–0.28)*	0.23 (0.12–0.33)*
Insufficient physical activity	− 0.03 (− 0.17–0.11)	0.22 (− 0.06–0.49)	− 0.02 (− 0.15–0.11)	− 0.13 (− 0.24- -0.03)*	− 0.1 (− 0.21–0.02)
Inadequate fruit intake	− 0.14 (− 0.29–0.01)	0.28 (− 01–0.66)	− 0.19 (− 0.34- -0.04)*	− 0.23 (− 0.34- -0.12)	−0.2 (− 0.33- -0.07)*
Inappropriate sugar intake	0.05 (− 0.09–0.19)	0.22 (− 0.13–0.57)	0.01 (− 0.13–0.16)	0.09 (− 0.03–0.21)	0.57 (0.34–0.81)*

*p < 0.05

Risk factors for diabetes (high BMI, and high waist circumference) were significant at the household and individual levels for both sexes across several measures of socio-economic status: education, income, social status, and area unsatisfactory basic needs index. A negative relationship between most measures of socioeconomic status (education, income SSI and UBNI) and fruit intake was observed in males.

Table 3 shows the comparison of the RII for diabetes mellitus and its risk factors among males and females by different measures of socio economic statuses. There were significant inequalities for prevalence of diabetes only in females at individual education level, household levels (i.e. household income and social status) and area unsatisfactory basic needs index. Here too there was significant inequalities for risk factors (high body mass index and high waist circumference) across most of the socioeconomic measures for both sexes (individual education levels, household income, social status index, area unsatisfactory basic needs index).

**Table 3:** Comparison of the relative index of inequality for diabetes mellitus and its risk factors among males and females by different measures of socio economic statuses

Morbidity / risk factor	Individual		Household		Area
	Education (95%CI)	Occupation (95%CI)	Income (95%CI)	Social status Index (95%CI)	Unsatisfactory Basic Needs Index (95%CI)
Male					
Prevalence of diabetes mellitus	1.85 (0.97–3.52)	0.39 (0.19–0.81)	2.5 (1.24–5.01)*	1.24 (0.69–2.21)	1.79 (0.99–3.26)
Prevalence of impaired glucose tolerance	1.87 (0.99–3.5)	0.35 (0.16–0.76)	1.28 (0.62–2.65)	0.88 (0.47–1.64)	1.4 (0.77–2.57)
High body mass index	2.25 (1.64–3.1)*	0.55 (0.39–0.78)	1.89 (1.30–2.74)*	1.56 (1.13–2.14)*	2.04 (1.46–2.85)*
High waist circumference	3.31 (2–5.47)*	0.35 (0.22–0.57)	3.97 (2.20–7.17)*	1.9 (1.15–3.16)*	3.21 (1.91–5.39)*
Family history of diabetes mellitus	1.88 (1.15–3.11)*	1.12 (0.52–2.39)	1.39 (0.75–2.58)	1.52 (0.96–2.4)	3.37 (1.93–5.88)*
Insufficient physical activity	1.08 (0.70–1.67)	0.60 (0.36–1.01)	1.34 (0.84–2.12)	1.0 (0.67–1.5)	1.13 (0.76–1.69)
Inadequate fruit intake	0.57 (0.46–0.70)	2.23 (1.4–3.56)*	0.61 (0.5–0.76)	0.68 (0.57–0.82)	0.62 (0.52–0.74)
Inappropriate sugar intake	0.99 (0.67–1.47)	0.76 (0.43–1.36)	1.31 (0.85–2.03)	1.01 (0.69–1.48)	1.05 (0.74–1.48)
Smoking^a^	0.65 (0.45–0.94)	2.63 (1.21–5.7)*	0.68 (0.47–0.98)	0.76 (0.55–1.04)	1.04 (0.76–1.41)
Alcohol consumption^a^	1.02 (0.81–1.27)	1.14 (0.8–1.64)	1.08 (0.86–1.35)	0.84 (0.7–1.02)	0.73 (0.62–0.87)
Female					
Prevalence of diabetes mellitus	2.3 (1.22–4.34)*	0.13 (0.04–0.41)	2.34 (1.24–4.43)*	2.37 (1.43–3.94)*	4.98 (2.71–9.15)*
Prevalence of impaired glucose tolerance	1.03 (0.53–1.97)	2.25 (0.23–22.5)	1.55 (0.82–2.92)	1.19 (0.72–1.95)	1.29 (0.77–2.16)
High body mass index	1.9 (1.46–2.47)*	0.38 (0.23–0.61)	1.61 (1.19–2.17)*	2.05 (1.66–2.55)*	2.61 (2.01–3.38)*
High waist circumference	2.04 (1.51–2.75)*	0.32 (0.19–0.54)	1.99 (1.42–2.79)*	2.02 (1.57–2.62)*	2.39 (1.79–3.19)*
Family history of diabetes mellitus	1.85 (1–3.42)	0.13 (0.03–0.51)	1.71 (0.86–3.40)	2.09 (1.25–3.5)*	3.34 (1.73–6.46)*
Insufficient physical activity	0.86 (0.53–1.4)	2.65 (0.53–13.18)	0.87 (0.53–1.43)	0.57 (0.38–0.85)*	0.69 (0.47–1.03)
Inadequate fruit intake	0.78 (0.6–1.01)	1.72 (0.77–3.84)	0.72 (056–0.94)	0.67 (0.55–0.81)	0.71 (0.57–0.88)
Inappropriate sugar intake	1.14 (0.75–1.72)	2.07 (0.61–7.05)	1.07 (0.7–1.62)	1.32 (0.94–1.86)	1.39 (0.98–1.96)

*p < 0.05

Table 4 describes the comparison of the concentration indexes for diabetes mellitus and its risk factors by different measures of socio economic status. Only males were included for the sections on smoking and alcohol because female consumption rates are almost nil. The concentration index is positive for prevalence of diabetes mellitus across household income for both sexes.

**Table 4:** Comparison of the concentration indexes for diabetes mellitus and its risk factors by different measures of socio economic statuses

Morbidity / risk factor	Individual		Household		Area
	Education (95%CI)	Occupation (95%CI)	Income (95%CI)	Social status Index (95%CI)	Unsatisfactory Basic Needs Index (95%CI)
Prevalence of diabetes mellitus	0.01 (−0.1–0.11)	0.12 (− 0.01–0.25)	0.03 (− 0.07–0.13)	0.05 (−0.05–0.16)	0.17 (0.07–0.26)*
Prevalence of impaired glucose tolerance	0.09 (−0.03–0.21)	0.07 (− 0.09–0.24)	0.09 (− 0.03–0.21)	0.07 (−0.05–0.19)	0.12 (0.1–0.24)*
High body mass index	0.23 (0.08–0.37)*	0.2 (− 0.01–0.4)	0.01 (− 0.13–0.16)	0.11 (−0.03–0.25)	0.19 (0.05–0.34)*
High waist circumference	0.14 (0.01–0.28)*	0.22 (0.05–0.39) *	0.03 (− 0.11–0.16)	0.12 (− 0.02–0.26)	0.23 (0.1–0.37)*
Family history of diabetes mellitus	0.06 (−0.07–0.18)	−0.001 (− 0.18–0.18)	0.05 (− 0.07–0.17)	0.06 (−0.05–0.16)	0.12 (0.01–0.23)*
Insufficient physical activity	0.11 (−0.02–0.23)	−0.18 (− 0.36- -0.002)*	0.02 (− 0.12–0.15)	0.01 (− 0.12–0.14)	−0.15 (− 0.27- -0.02)*
Inadequate fruit intake	−0.14 (− 0.28- -0.003)*	− 0.14 (− 0.33–0.04)	−0.29 (− 0.43- -0.15)*	− 0.41 (− 0.54- -0.28)*	− 0.37 (− 0.5- -0.24)*
Inappropriate sugar intake	0.20 (0.07–0.34)*	0.13 (− 0.07–0.33)	0.28 (0.14–0.42)*	0.33 (0.2–0.46)*	0.46 (0.33–0.58)*
Smoking^a^	− 0.12 (− 0.35–0.11)	− 0.31 (− 0.56- -0.07)*	− 0.04 (− 0.27–0.18)	− 0.06 (− 0.29–0.16)	0.16 (− 0.07–0.39)
Alcohol consumption^a^	− 0.01 (− 0.21–0.19)	−0.07 (− 0.28–0.15)	0.01 (− 0.19–0.21)	0.21 (0.001–0.42)*	0.19 (− 0.02–0.39)

*p < 0.05

^a^Smoking and Alcohol consumption was tested only for males

Insufficient physical activity, alcohol intake and sugar intake did not show a consistent relationship across most of the socio-economic status of males and females. Too much emphasis cannot be attributed to family history of diabetes because it is not a modifiable risk factor and relatively well analyzed in the existing literature.

## Discussion

The results show the presence of a variable relationship between socioeconomic status and prevalence of diabetes and its risk factors, depending on the measures of inequalities used. However, there is a relatively consistent relationship observed where prevalence of diabetes is higher in those with better individual levels of education and occupation, and higher household incomes. The prevalence of risk factors (high BMI, and high WC) for both sexes is also significantly higher in those with better individual education and higher socio-economic status (i.e. using household income, social status index and area unsatisfactory basic needs index - SII and RII). Inadequate fruit intake and smoking (among males) demonstrated a pro-poor relationship indicating adverse dietary habits in these groups.

Early literature from high income countries reported a pattern where the affluent were at high risk of diabetes [46] In comparison, more recent literature from these countries report higher rates of diabetes mellitus and its risk factors in the poorer population groups [16, 47–52].

This suggests that during economic transitions adverse health behaviors are initially encountered in the higher socioeconomic sector and are later transmitted to the lower socioeconomic groups [53]. It may also be that higher socioeconomic categories modify their risky behavior early while lower socioeconomic categories persist with the adverse health behaviors during the course of a country’s economic development [53]. It is possible that similar factors are operating in Sri Lanka. The pro rich pattern of diabetes and its risk factors and the relatively small magnitude of the slope index seen in the present study may suggest that the country is in an economic transitional stage [45].

The inequality also seemed to vary depending on the sex of the individual. The inequalities for the prevalence of diabetes mellitus were more marked for females possibly due to the gender differences in health behavior, as shown by the correspondingly reduced physical activity and inadequate fruit intake in females. Similar observations were made for females in Europe on the prevalence of diabetes mellitus and obesity [16, 48, 54], where these were attributed to the corresponding gender differences in health behaviors; it is possible these differences are exaggerated in South Asian or lower middle income settings. Females also had a higher SII and RII for diabetes mellitus and its risk factors in relation to household level SSI compared to males. This may possibly be due to the females’ enhanced role in social networking compared to males in this study setting, as it was included for the assessment of SSI.

The study suggests that measures to prevent diabetes should target specific factors based on sex and socio economic status rather than implementing interventions as blanket coverage and helps identify such areas and groups.

The weakest relationship between prevalence of diabetes and its risk factors in relation to socioeconomic status was observed with the concentration index. The inequality measures are known to be influenced by extreme wealth/poverty and less extreme incomes [55, 56]. Therefore the performance of these inequity measures in LIC/LMICs requires further investigation.

Our study is unique as it assesses the inequalities of diabetes mellitus and its risk factors in a district of a lower middle income country with free healthcare for all at the point of delivery. However, we did not explore possible interactions between each of these socioeconomic indicators. Therefore we cannot speculate on the effect of a change in the inequality of a single socioeconomic indicator, on the distribution of diabetes mellitus and its risk factors. Further the study dichotomized alcohol consumption and smoking (i.e. alcohol users or smokers as one or more glasses of alcohol or smoked at least once during the past fortnight). This fails to capture the extent of smoking or alcohol across socio-economic groups. Measuring prevalence alone may not fully represent inequalities in the impact of the studied health risks; the health impact of the variable level of risk by each risk factor may need to be explored. As patterns of distribution of risk factors over time are not seen in this study, further longitudinal studies are needed to track these changes and to understand the drivers of these trends. Other diabetes associated risk factors and diseases such as lipid levels and hypertension were not included in the present study, but may show inequalities in their distribution. Future studies may include a broader range of risk factors and non-communicable diseases to provide a more comprehensive distribution of risk profiles across different socioeconomic groups.

This study points to the importance of looking at multiple socioeconomic indicators when examining disparities and implies a specificity of mechanisms that link health inequality to socioeconomic indicators.

## Conclusion

The results show a variable relationship between socioeconomic status and prevalence of diabetes and its risk factors. The inequalities in the prevalence of diabetes and risk factors vary depending on gender and the measure of inequality used. The weakest relationship between prevalence of diabetes and its risk factors in relation to socioeconomic status was observed with the concentration index.

The observations together with the trajectories of prevalence in other countries suggest that Sri Lanka is in a period of economic transition, and that poorer groups may develop relatively higher rates of diabetes, obesity and risk factors in the future.

The study suggests that measures to prevent diabetes should focus on targeting specific factors based on sex and socio economic status. The priority target areas for interventions should include prevention of obesity (BMI and central obesity) specifically in more affluent females. Males who have a low level of education and in non-skilled occupations should be especially targeted to reduce smoking and increase fruit intake.

## Abbreviations

DM: Diabetes Mellitus
RII: Relative Index of Inequality
SII: Slope Index of Inequality
SSI: Social Status Index
UBNI: Unsatisfactory Basic Needs Index
